# RACIAL AND ETHNIC DIFFERENCES IN THE LIFE COURSE INFLUENCES OF SOCIOECONOMIC STATUS ON ADULT SYSTEMIC INFLAMMATION

**DOI:** 10.1093/geroni/igac059.1636

**Published:** 2022-12-20

**Authors:** Amber Kelly, Patricia Morton, Fatima Eid, Kenneth Ferraro

**Affiliations:** Wayne State University, Ferndale, Michigan, United States; Wayne State University, Detroit, Michigan, United States; Wayne State University, Dearborn, Michigan, United States; Purdue University, West Lafayette, Indiana, United States

## Abstract

Two fundamental causes of health disparities in the U.S. are race and socioeconomic status (SES). Although these disparities emerge early in the life course, there is limited research on how race/ethnicity intersects with SES over the life course to influence later-life systemic inflammation—a key pathogenic process of health for older adults. Moreover, life course studies on systemic inflammation often focus on Black/White differences. Analyzing a sample of Black, White, and Hispanic Americans, this study examined the intersecting influences of childhood SES, race/ethnicity, and adult education on three time points of later-life inflammation levels (measured using C-reactive protein [CRP]). Longitudinal mixed-effects models were estimated using a sample of U.S. men (n=4,137) and women (n=6,187) over age 50 from the Health and Retirement Study during a decade of observation (2006-2016). Black men who experienced low childhood SES had higher levels of CRP than White men with low childhood SES; education did not moderate these associations. Education had a stronger protective effect against systemic inflammation for Hispanic men who experienced childhood socioeconomic disadvantage compared to their White counterparts. For women, the protective effect of education on systemic inflammation was stronger for White women compared to Black and Hispanic women. There were no significant childhood SES interactions for women. Findings indicate that the effects of SES on later-life systemic inflammation are contingent upon race/ethnicity, suggesting that intersectionality operates over the life course to produce health disparities in later-life. However, adult education acts as a leveler of life course inequality for Hispanic men.

